# Combination therapy with HSP90 inhibitors and piperlongumine promotes ROS-mediated ER stress in colon cancer cells

**DOI:** 10.1038/s41420-023-01672-y

**Published:** 2023-10-13

**Authors:** Chenyu Qiu, Xin Shen, Hui Lu, Yinghua Chen, Chenxin Xu, Peisen Zheng, Yiqun Xia, Junqi Wang, Yafei Zhang, Shaotang Li, Peng Zou, Ri Cui, Jundixia Chen

**Affiliations:** 1 Affiliated Yueqing Hospital, Wenzhou Medical University, Wenzhou, 325035 China; 2 School of Pharmaceutical Sciences, Wenzhou Medical University, Wenzhou, 325035 China; 3 The First Affiliated Hospital of Wenzhou Medical University, Wenzhou Medical University, Wenzhou, 325035 China

**Keywords:** Colorectal cancer, Cell death, Endoplasmic reticulum

## Abstract

Colon cancer is a major cause of cancer-related death. Despite recent improvements in the treatment of colon cancer, new strategies to improve the overall survival of patients are urgently needed. Heat shock protein 90 (HSP90) is widely recognized as a promising target for treating various cancers, including colon cancer. However, no HSP90 inhibitor has been approved for clinical use due to limited efficacy. In this study, we evaluated the antitumor activities of HSP90 inhibitors in combination with piperlongumine in colon cancer cells. We show that combination treatment with HSP90 inhibitors and piperlongumine displayed strong synergistic interaction in colon cancer cells. These agents synergize by promoting ER stress, JNK activation, and DNA damage. This process is fueled by oxidative stress, which is caused by the accumulation of reactive oxygen species. These studies nominated piperlongumine as a promising agent for HSP90 inhibitor-based combination therapy against colon cancer.

## Introduction

Colon cancer is one of the leading causes of cancer-related deaths worldwide, and its incidence and mortality are rising [1]. Surgical resection is the main method in the treatment of colon cancer, and is the first choice for early-stage colon cancer. However, it is not suitable for most metastatic tumors [2]. Most patients with colon cancer are in the late-stage when they are diagnosed for the first time. Consequently, chemotherapy has a vital role in the treatment of advanced colon cancer. Some cytotoxic agents, such as irinotecan, oxaliplatin and fluoropyrimidines, have increased the survival rate of patients with advanced colon cancer [3, 4]. However, systemic side effects and drug resistance limited the clinical application of these agents [5, 6]. Therefore, more effective treatments for colon cancer are urgently needed.

HSP90 is a molecular chaperone that promotes the folding and stability of its substrate proteins in cells [7, 8]. HSP90 is upregulated in numerous cancers, which is thought to facilitate the maturation of numerous oncoproteins and promote tumor growth [9, 10]. Therefore, HSP90 is widely recognized as a promising target for treating various cancers [11, 12]. Many HSP90 inhibitors have been discovered, and some agents have entered clinical trials [13, 14]. Tanespimycin (17-AAG), one of these agents, was the first HSP90 inhibitor to enter clinical trials, and has shown clinical activity in various human cancers [15, 16]. However, poor solubility and limited bioavailability limit its efficacy in clinical trials. Hitherto, no HSP90 inhibitor can satisfy the clinical requirement due to limited efficacy, but much stronger anticancer efficacy has been achieved when they are used in combination with chemotherapies or targeted agents [17–19].

Reactive oxygen species (ROS) are by-products of aerobic metabolism [20]. Oncogenic stimulation and increased metabolic activity lead cancer cells frequently exhibit increased levels of ROS than normal cells, which renders cancer cells more vulnerable to oxidative stress [21, 22]. Therefore, manipulating ROS levels in cancer cells might be an effective strategy to kill these cells [23–25]. Numerous studies have shown that radiation and some clinical drugs can induce cancer cell death by regulating intracellular redox homeostasis [26–28]. A previous study uncovered that doxorubicin can increase cellular ROS levels and induce apoptosis in colon cancer cells [29]. Moreover, the accumulation of ROS is also involved in the antitumor activity of cisplatin and bortezomib [30, 31].

## Results

### Combination of PL with HSP90 inhibitors synergistically suppresses cancer cell growth

**Fig. 1:**
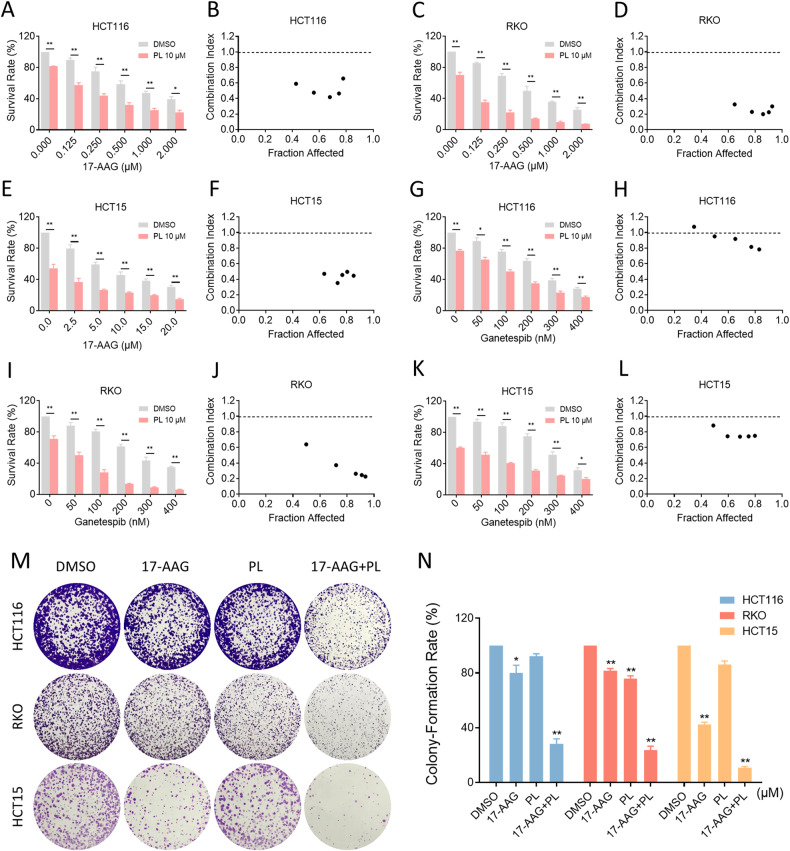
Combination of PL with HSP90 inhibitors suppressed cell proliferation. **A**–**F** Cell viability in HCT116, RKO, or HCT15 cells treated with the indicated concentration of 17-AAG or combined with PL for 24 h. **G**–**L** Cell viability in HCT116, RKO, or HCT15 cells treated with the indicated concentration of ganetespib or combined with PL for 24 h. CI values were calculated using CompuSyn software. **M**, **N** Colony-forming ability of HCT116, RKO, or HCT15 cells. The concentration of 17-AAG on HCT116, RKO, and HCT15 cells was 0.0625 μM, 0.03125 μM and 1.25 μM, respectively, and the concentration of PL was 1.25 μM all the same. (*p < 0.05, **p < 0.01).

We first evaluated the synergistic effect of PL and HSP90 inhibitors in colon cancer cells. As shown in Fig. 1A–L, HSP90 inhibitors 17-AAG and ganetespib reduced the viability of colon cancer cells in a dose-dependent manner. Notably, the growth suppressive effect was more evident when HSP90 inhibitors were combined with PL. According to the analysis of combination index (CI) values, a synergistic effect with CI < 1 was observed in almost all combination methods, indicating a significant synergy between HSP90 inhibitors and PL. In addition, 17-AAG displayed a better synergistic effect with PL than ganetespib (Fig. 1A–L). Therefore, the combination of 17-AAG with PL was further evaluated for its effect on the colony forming ability of colon cancer cells. As shown in Fig. 1M, N, 17-AAG in combination with PL resulted in a significant decline in colony numbers.

### Combination of PL with 17-AAG increases ROS production and causes DNA damage in colon cancer cells

**Fig. 2:**
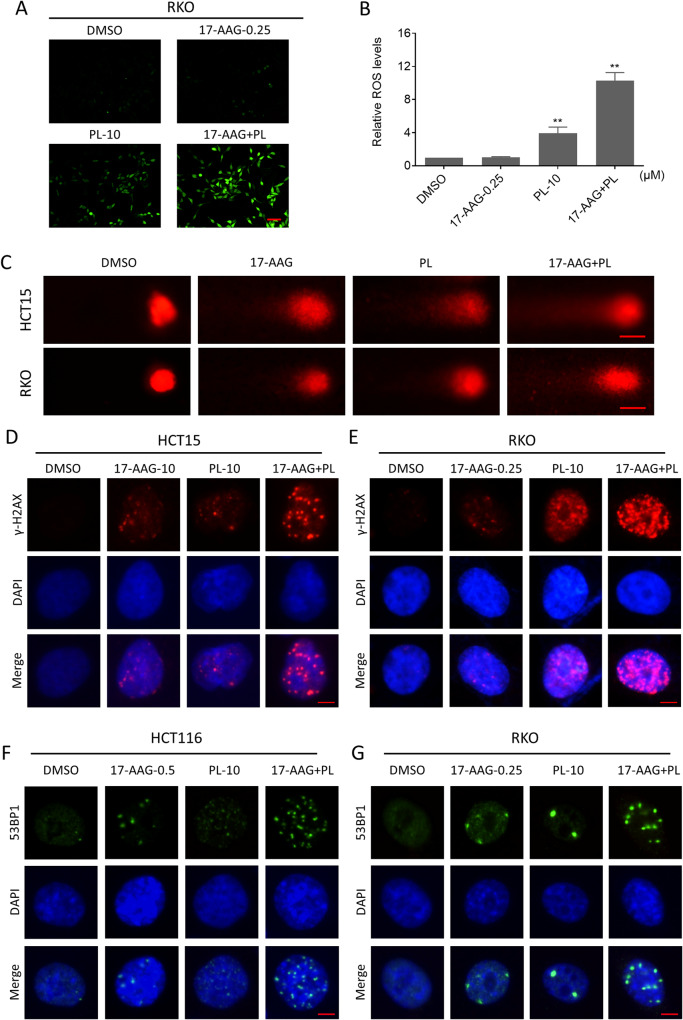
Combination of PL with 17-AAG increased ROS production and induced DNA damage. **A**, **B** Intracellular ROS levels in RKO cells treated with 17-AAG (0.25 μM) or PL (10 μM) or their combination for 2 h (**p < 0.01). Scale bar = 75 µm. **C** Representative images of cell trailing in a comet assay. Scale bar = 10 µm. **D**, **E** Representative images taken by fluorescence microscopy showing nuclear foci formation of γ-H2AX in HCT15 or RKO cells. Scale bar = 5 µm. **F**, **G** Representative images taken by fluorescence microscopy showing nuclear foci formation of 53BP1 in HCT116 or RKO cells. Scale bar = 5 µm.

Previous research has suggested that PL exerts antitumor activity by upregulating ROS levels [32, 33]. However, the effect of PL combined with 17-AAG on ROS levels remains unknown. Therefore, we detected the level of ROS after the combination treatment. Indeed, PL treatment alone induced ROS generation, but a greater increase in ROS levels was achieved when it was combined with 17-AAG (Fig. 2A, B). Excessive accumulation of ROS causes oxidative damage to DNA, leading to cell death [34, 35]. Therefore, we investigated the DNA damage of cells after exposure to the compounds. We first performed a comet assay, in which damaged DNA exhibits the shape of a comet tail under an electric field, and the length of the tail was positively correlated with the degree of DNA damage. The results showed that the combination therapy group had more severe cellular tail compared to the monotherapy group, indicating more severe DNA damage (Fig. 2C). In addition, we detected the expression and distribution of DNA damage-related indicators γ-H2AX and 53BP1 by immunofluorescence assay. The results showed that 17-AAG in combination with PL caused an obvious increase of nuclear γ-H2AX foci in colon cancer cells (Fig. 2D, E). Similarly, the combination also significantly increased the nuclear 53BP1 foci in colon cancer cells, which also reflected the severity of DNA damage due to the increased DNA repair sites (Fig. 2F, G).

### ROS scavenger resolves DNA damage and cell viability inhibition caused by combination treatment

**Fig. 3:**
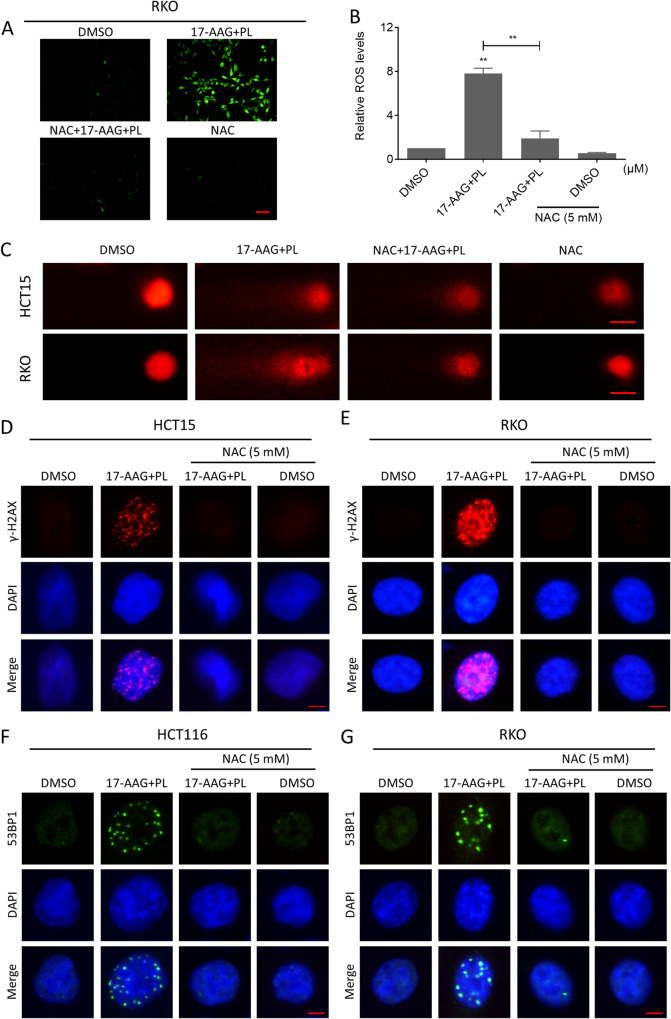
ROS scavenger reversed the efficacy of the combination treatment. **A**, **B** Intracellular ROS levels in RKO cells treated with the combination of 17-AAG and PL for 2 h following pretreatment with NAC (5 mM) for 1 h (**p < 0.01). Scale bar = 75 µm. **C** Representative images of cell trailing in a comet assay. Scale bar = 10 µm. **D**, **E** Representative images showing nuclear foci formation of γ-H2AX in HCT15 or RKO cells treated with the combination of 17-AAG and PL following pretreatment with NAC (5 mM) for 1 h. Scale bar = 5 µm. **F**, **G** Representative images showing nuclear foci formation of 53BPl in HCT116 or RKO cells treated with the combination of 17-AAG and PL following pretreatment with NAC (5 mM) for 1 h. Scale bar = 5 µm.

**Fig. 4:**
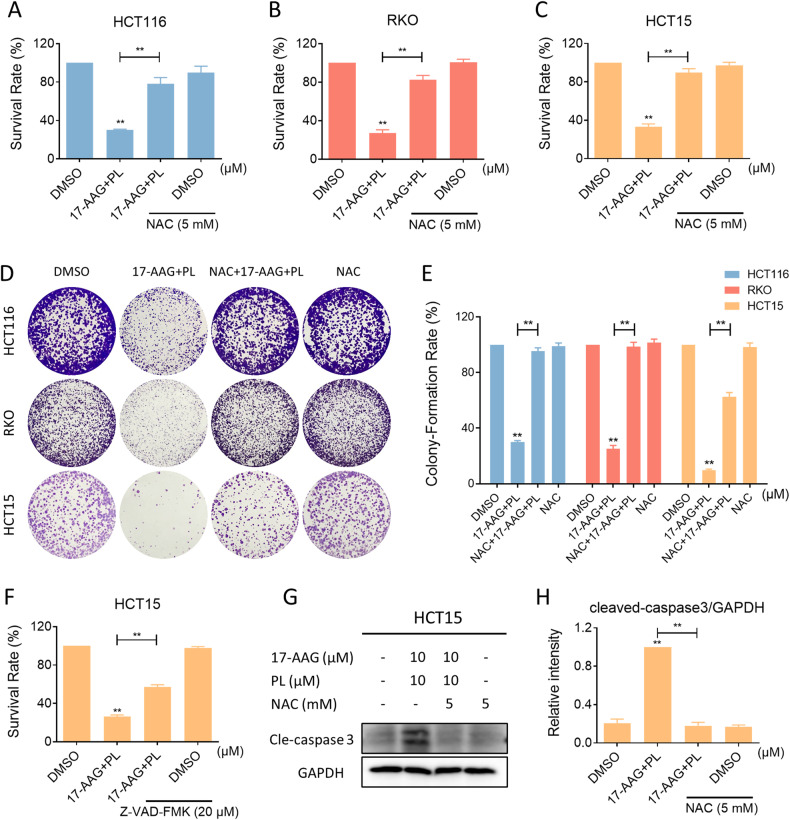
ROS scavenger reversed inhibition of cell viability by combination treatment. **A**–**C** Cell viability in HCT116, RKO, or HCT15 cells treated with the combination of 17-AAG and PL for 24 h following pretreatment with NAC (5 mM) for 1 h. **D**, **E** Colony-forming ability of HCT116, RKO, or HCT15 cells after pretreatment with NAC (5 mM). The concentration of 17-AAG on HCT116, RKO, and HCT15 cells was 0.0625 μM, 0.03125 μM and 1.25 μM, respectively, and the concentration of PL was 1.25 μM all the same. **F** Cell viability in HCT15 cells treated with the combination of 17-AAG and PL for 24 h following pretreatment with Z-VAD-FMK (20 μM) for 1 h. **G**, **H** The expression of cleaved-caspase-3 in HCT15 cells was detected by western blot (*p < 0.05, **p < 0.01).

To further demonstrate that the elevated ROS levels were responsible for DNA damage and cell death, we pretreated the cells with N-Acetyl-L-cysteine (NAC), an ROS scavenger, before combination treatment. The accumulation of intracellular ROS levels in colon cancer cells was markedly reversed after NAC pretreatment (Fig. 3A, B). As a result, the cellular tail phenomenon and the foci of DNA damage-related markers, γ-H2AX or 53BP1 in the nucleus, were also reversed (Fig. 3C–G). Importantly, NAC eliminated the cooperative effects of 17-AAG and PL on cytotoxicity and inhibition of colony forming ability (Fig. 4A–E). To investigate the mechanism by which the combination treatment causes cell death, we utilized two inhibitors of the cell death pathway and found that only the Z-VAD-FMK, the pan-caspase pathway inhibitor, significantly reversed the cell death caused by the combination treatment (Fig. 4F). Furthermore, the combination treatment caused an increase in cleaved-caspase-3 expression in HCT15 cells, which was reversed by pre-treating with NAC (Fig. 4G, H). Our results suggested that the accumulation of ROS is required for the synergism between 17-AAG and PL, and this effect may be responsible for cell death by inducing apoptosis.

### Combination of PL with 17-AAG activates the JNK signaling pathway

**Fig. 5:**
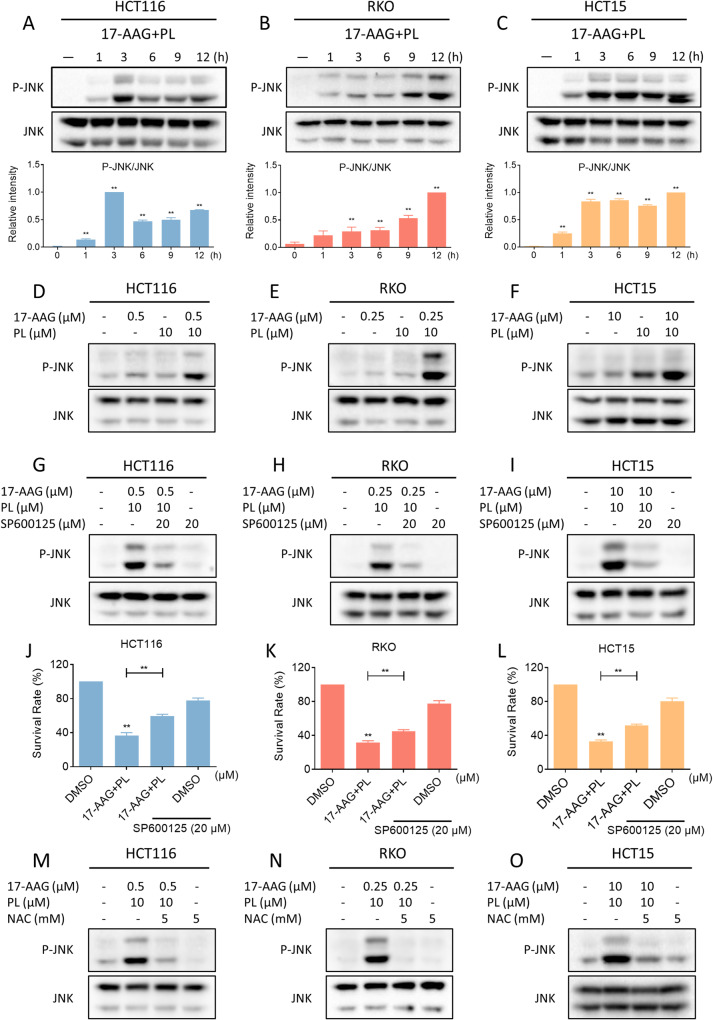
Combination of PL with 17-AAG activated JNK signaling pathway. **A**–**C** The expression of phosphorylated JNK and JNK in HCT116, RKO, or HCT15 cells was detected by western blot (**p < 0.01). **D**–**F** The expression of phosphorylated JNK and JNK in HCT116, RKO, or HCT15 cells treated with 17-AAG or PL alone or their combination. **G**–**I** The expression of phosphorylated JNK and JNK in HCT116, RKO, or HCT15 cells was detected by western blot. **J**–**L** Cell viability in HCT116, RKO, or HCT15 cells treated with the combination of 17-AAG and PL for 24 h following pretreatment with SP600125 (20 μM) for 1 h. **M**–**O** Western blot analysis of phosphorylated JNK and JNK in HCT116, RKO, or HCT15 cells pretreated with NAC (5 mM) for 1 h before the combination treatment.

The c-Jun N-terminal kinase (JNK) signaling pathway is normally activated in ROS-dependent cell death [36, 37]. We, therefore, examined the effect of combination therapy on the expression of p-JNK, and the results showed that 17-AAG in combination with PL markedly increased the expression of p-JNK in colon cancer cells (Fig. 5A–C). Notably, 17-AAG and PL together resulted in a more significant increase in the JNK phosphorylation than either treatment alone (Fig. 5D–F). To confirm whether JNK was related to the synergism between 17-AAG and PL, we blocked JNK activity by using SP600125, a specific inhibitor of JNK. As shown in Fig. 5G–I, pretreatment with SP600125 significantly suppressed the expression of p-JNK in drug-treated cells. Importantly, the synergism between 17-AAG and PL in cell growth inhibition was partially blocked by SP600125, demonstrating that activation of the JNK pathway is essential for the synergism between 17-AAG and PL (Fig. 5J–L). Moreover, pretreatment with NAC significantly blocked the JNK pathway activation, suggesting that JNK is a downstream effector of ROS-mediated cell death (Fig. 5M–O).

### PL and 17-AAG cooperate to induce ER stress in colon cancer cells

**Fig. 6:**
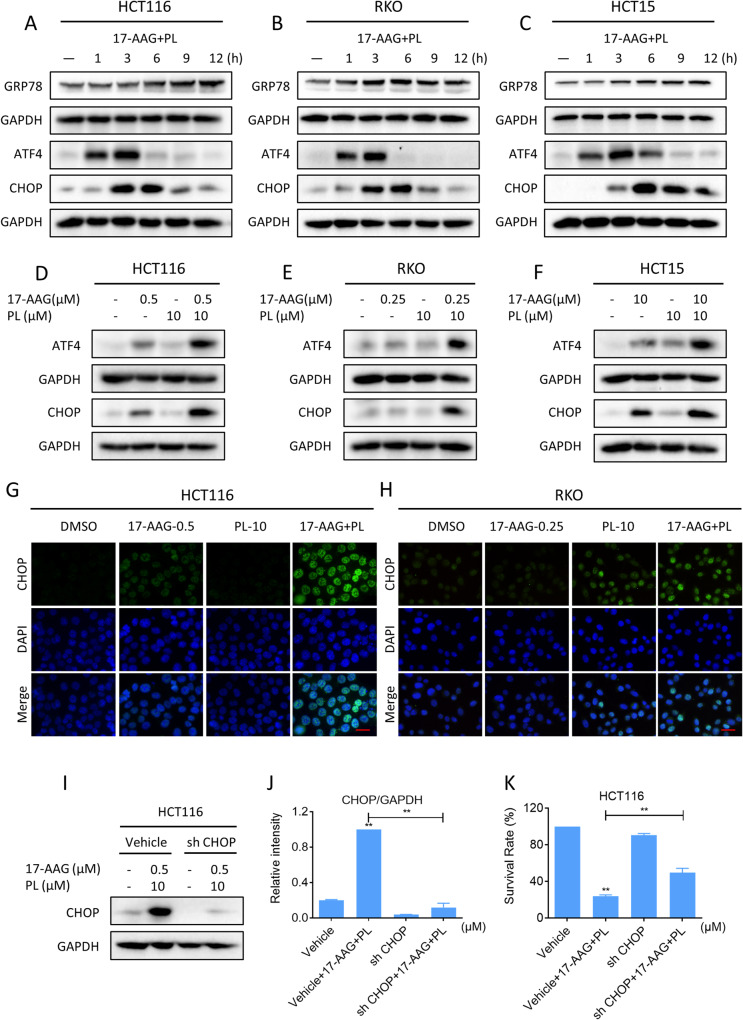
PL and 17-AAG synergize to induce ER stress in colon cancer cells. **A**–**C** The expression of GRP78, ATF4, and CHOP in HCT116, RKO, or HCT15 cells was detected by western blot. **D**–**F** The expression levels of ATF4 and CHOP in HCT116, RKO, or HCT15 cells. **G**, **H** Representative images showing the expression of CHOP in nuclear within HCT116 or RKO cells treated with 17-AAG or PL alone or their combination. Scale bar = 25 µm. **I**, **J** The expression of CHOP in HCT116 cells infected with recombinant lentivirus after 6 h of combination treatment. **K** The cell viability of HCT116 cells infected with recombinant lentivirus after 24 h of combination treatment was measured by trypan blue dye exclusion assay (**p < 0.01).

The molecular chaperone HSP90 helps its substrate proteins to fold and stabilize, thereby regulating a variety of tumor signaling pathways [8, 38]. Inhibition of HSP90 by specific inhibitors is known to trigger endoplasmic reticulum (ER) stress in various cancer cells [39, 40]. We then investigated whether 17-AAG and PL synergize to induce ER stress in colon cancer cells. As shown in the results, increased expression of GRP78, ATF4, and CHOP was detected in colon cancer cells after combination therapy. Notably, 17-AAG combined with PL treatment resulted in a greater increase in the expression of ATF4 and CHOP compared to either treatment alone (Fig. 6A–F). Further analysis by immunofluorescence revealed that the accumulation of CHOP was concentrated in the nucleus (Fig. 6G, H). To confirm whether CHOP was required for the synergistic effect of 17-AAG and PL, the expression of CHOP was knocked down using lentivirus system (Fig. 6I, J). Notably, knockdown of CHOP significantly reversed the synergism between 17-AAG and PL in cell growth inhibition, indicating that CHOP is critical for the synergistic effect (Fig. 6K).

**Fig. 7:**
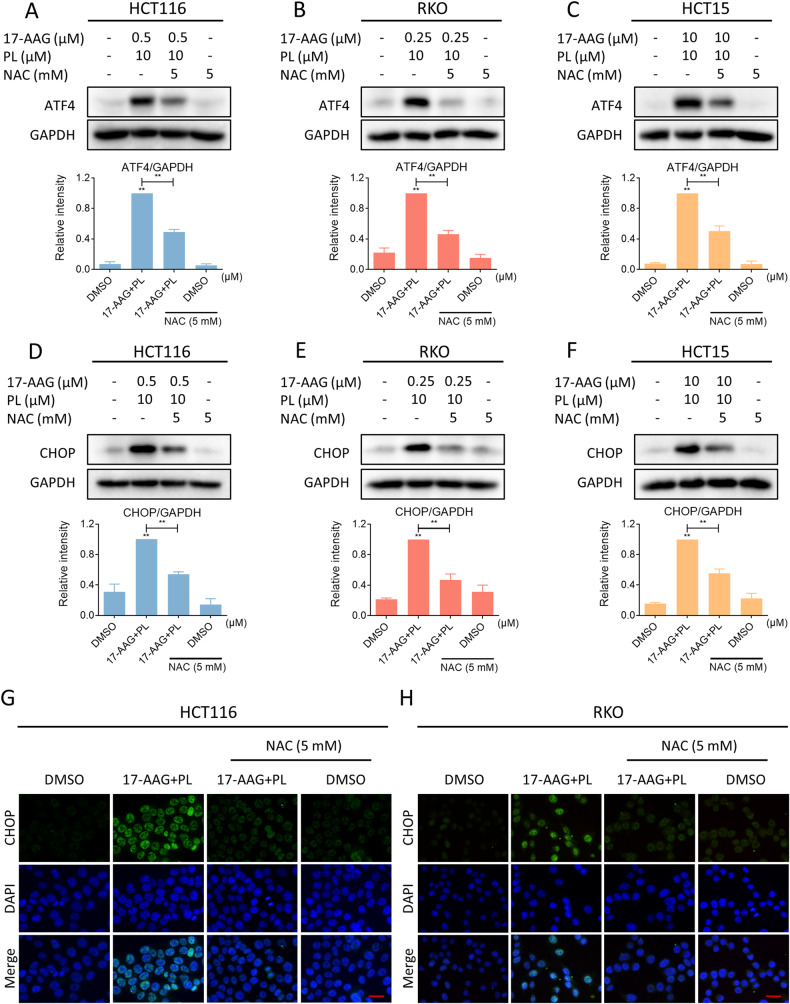
ROS scavenger blocked the activation of ER stress. **A**–**C** Cells were pretreated with NAC (5 mM) for 1 h before being treated with a combination of 17-AAG and PL, and the expression of ATF4 in HCT116, RKO, or HCT15 cells was detected by western blot (**p < 0.01). **D**–**F** The expression of CHOP in HCT116, RKO, or HCT15 cells. **G**, **H** Representative images showing expression of CHOP in nuclear within HCT116 or RKO cells were taken using a fluorescence microscope. Scale bar = 25 µm.

Recently, emerging studies have demonstrated that excessive ROS accumulation can cause oxidative stress and trigger ER stress [41, 42]. Therefore, we asked whether there is a possible link between ROS and ER stress in combination-treated cells. We pretreated NAC to block ROS generation, and then we detected a significant reversal of the increased expression of ATF4 and CHOP. (Fig. 7A–F). Moreover, immunofluorescence assay results indicated that NAC pretreatment significantly reversed the accumulation of CHOP in the nucleus (Fig. 7G, H). These results support the view that ROS accumulation can trigger ER stress and CHOP-dependent cell death.

### PL and 17-AAG synergize to inhibit the growth of colon cancer cells in vivo

**Fig. 8:**
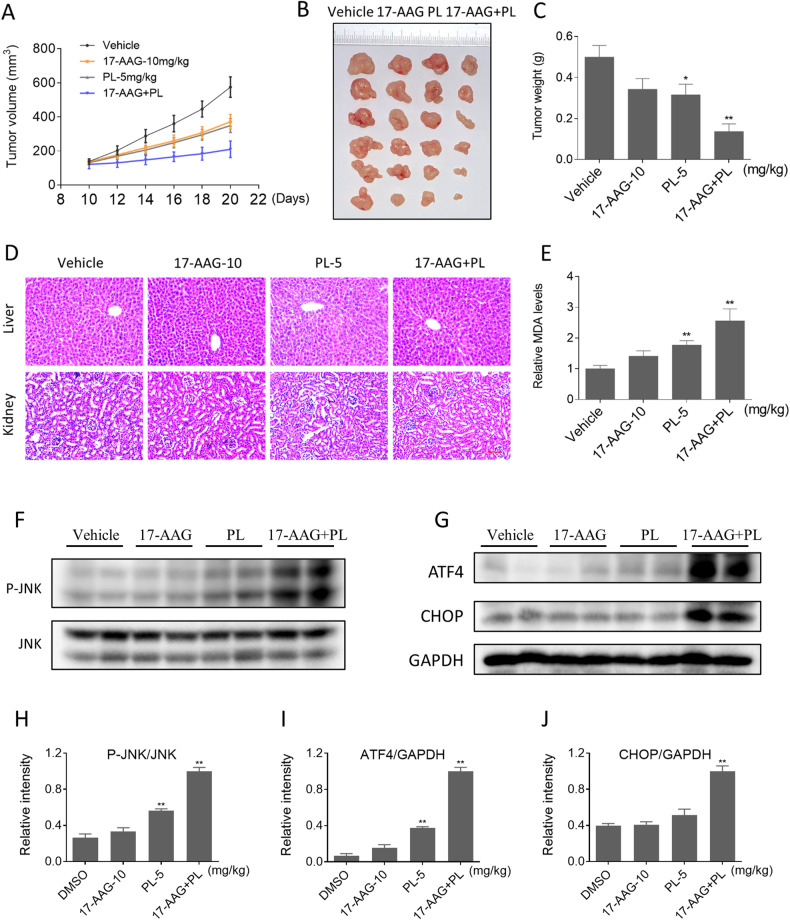
PL and 17-AAG synergize to inhibit the growth of colon cancer cells in vivo. **A**–**C** Combination treatment of 17-AAG (10 mg/kg) and PL (5 mg/kg) greatly inhibited tumor volume and weight (*p < 0.05, **p < 0.01). **D** HE staining of major organs. Scale bar = 75 µm. **E** The relative expression of MDA in tumor tissues. **F**–**J** The expression levels of p-JNK, ATF4, and CHOP were detected by western blot.

We further explored the effect of the combination of PL and 17-AAG on tumor growth in vivo. As shown in Fig. 8A–C, 17-AAG (10 mg/kg) in combination with PL (5 mg/kg) resulted in a greater reduction in tumor growth than either treatment alone. Histopathological analysis of major organs showed that the combination treatment did not induce any significant toxicity (Fig. 8D). Malondialdehyde (MDA) is an active aldehyde produced as a result of lipid peroxidation and serves as a valuable biomarker for oxidative stress. Indeed, the MDA levels were obviously increased in the combination treatment group (Fig. 8E). Similarly, we detected the JNK pathway and the expression of ATF4 and CHOP in tumor tissues. Consistent with the in vitro results, 17-AAG combined with PL increased JNK phosphorylation and the expression of ATF4 and CHOP (Fig. 8F–J). These results suggest that the combination of 17-AAG and PL is a promising therapeutic approach for colon cancer.

## Discussion

**Fig. 9:**
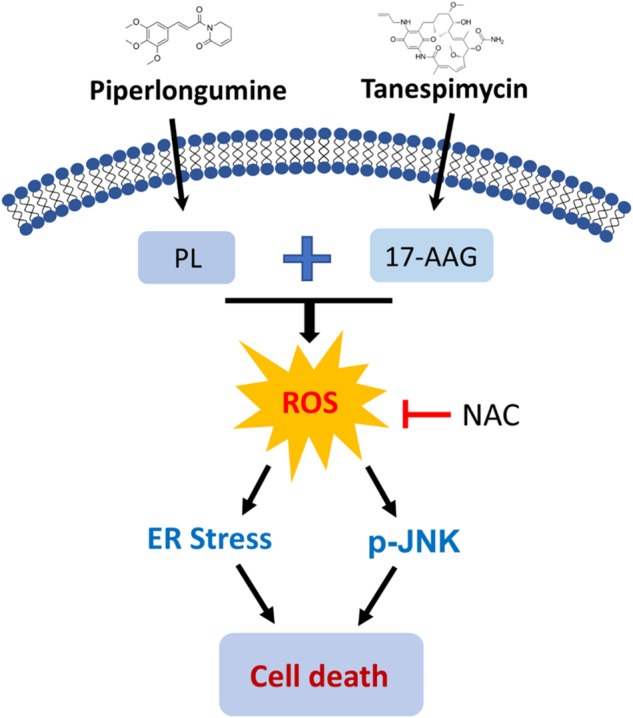
The proposed working model.

Treatment of colon cancer has always been a challenging issue. HSP90 is a molecular chaperone that is overexpressed in a variety of cancers and plays a key role in the folding and stabilization of oncogenic proteins [12, 43]. In recent years, numerous HSP90 inhibitors have been discovered and some agents have entered clinical trials [13]. However, no HSP90 inhibitors have been approved by the FDA due to their limited efficacy [44]. Research on HSP90 and its inhibitors has not stopped. Although the therapeutic effect of HSP90 inhibitors alone is limited, the prospect of HSP90 inhibitors in combination with other drugs deserves much attention. Administering HSP90 inhibitors to cancer cells weakens their ability to cope with stress. Therefore, it is possible to achieve a better therapeutic effect by combining with other ROS-inducing agents. At the beginning of this study, we examined the synergistic inhibition of colon cancer cell proliferation by PL and two HSP90 inhibitors, 17-AAG and ganatespib, and found that PL potentiated the effects of both HSP90 inhibitors. 17-AAG and ganatespib inhibit the ATPase activity of HSP90, consequently impacting its function [45], both of them entered clinical studies, however failed with limited efficacy [46, 47]. Although some studies have shown that ganatespib has better effects and lower toxicity compared to 17-AAG [48], in our study we showed that the synergistic effect of ganatespib with PL was not as good as that of 17-AAG. Therefore, we chose 17-AAG in this study to investigate the effect and mechanism of its synergistic effect with PL. We found that PL could greatly improve the anticancer efficacy of 17-AAG in colon cancer cells. The synergistic effects of PL with 17-AAG were dependent on ROS generation, ER stress induction, and JNK signaling pathway activation (Fig. 9). Importantly, the combination of PL and 17-AAG demonstrated significant inhibition of tumor growth in vivo, indicating that this combination is a promising therapeutic option for colon cancer.

High ROS levels are widely considered to be a general physiological manifestation of cancer cells, and cancer cells are more likely to reach the toxic threshold of ROS and are more sensitive to redox imbalances than normal cells. Therefore, disrupting the balance of redox states is a promising strategy to selectively eliminate these cells [24, 49]. Upregulation of ROS levels and depletion of antioxidant capacity are ways to disrupt the redox balance. Mitochondria are usually considered to be the main producers of ROS. However, this is not the only way in which ROS are upregulated, when the antioxidant capacity of the cell is reduced, ROS are also upregulated. Many peroxidases, such as glutathione peroxidase and thiol peroxidase, are capable of exerting antioxidant capacity, and when their activity is reduced, the redox balance in the cell becomes unstable and thus susceptible to oxidative stress [50–52]. Recent data have revealed that some anticancer drugs can induce cancer cell death by upregulating ROS levels in their specific ways [27, 53]. PL, a ROS generator, has been implicated in the generation of ROS. We here further observed that PL in combination with 17-AAG significantly enhanced the accumulation of ROS in colon cancer cells. Importantly, the synergism between PL and 17-AAG in inhibiting cell growth was markedly abolished by NAC, indicating that the accumulation of ROS is crucial for this combination. These findings further support the view that manipulating ROS levels in cancer cells is a promising strategy for treating cancer. However, further experiments are needed to explore the detailed mechanism by which these two compounds synergistically upregulate ROS in colon cancer cells.

Previous studies have shown that ROS is an upstream mediator of ER stress-induced cell death [54, 55]. Moreover, increased phosphorylation of JNK is also a manifestation of stress caused by excessive ROS [56]. As expected, we observed that PL and 17-AAG synergistically increased ROS levels, thereby activating ER stress and JNK signaling pathways to mediate colon cancer cell death. Importantly, the combination therapy was more effective in activating these cell death pathways than the single treatment. In addition, our results identified that the cooperative effect of PL and 17-AAG on cell death was partially reversed by CHOP knockdown or JNK inhibitor. Considering that CHOP knockdown or JNK inhibitor can only partially reverse the combined effect, indicating that there may be other signaling pathways mediating the synergistic effect of PL and 17-AAG, and further experiments are needed to address this issue.

Our results demonstrated that PL could potentiate the antitumor efficacy of HSP90 inhibitors by inducing ROS generation. Our research provides a useful strategy for combining HSP90 inhibitors with existing ROS inducers or physical treatments. In addition, our studies identified a novel therapeutic combination for colon cancer. However, to provide a solid theoretical basis for the use of this combination in clinical therapy, more detailed in vivo pharmacokinetics need to be confirmed.

## Methods

### Materials

Tanespimycin (17-AAG, T6290), ganetespib (T2309), piperlongumine (PL, T6947), SP600125 (T3109) and Z-VAD-FMK (T7020) were purchased from Targetmol (Boston, USA). 17-AAG, ganetespib, PL and SP600125 were dissolved in DMSO, and then divided into small tubes and stored at −80 °C. Final DMSO content ≤ 0.1% when these drug solutions were used. Antibodies including phospho-SAPK/JNK (1:1000, 4668 S), SAPK/JNK (1:1000, 9252 S), ATF4 (1:1000, 11815 S), γ-H2AX (1:200, 9718 S) and cleaved-caspase-3 (1:1000, 9661 S) were purchased from Cell Signaling Technology (Danvers, USA). CHOP (1:1000, 15204-1-AP), GAPDH (1:20000, 10494-1-AP), GRP78 (1:2000, 11587-1-AP) and secondary antibody (1:4000, SA00001-2) were purchased from Proteintech Group (Wuhan, China). 53BP1 (1:800, NB100-304) was obtained from Novus Biologicals (Littleton, USA).

### Cell culture

HCT116, RKO and HCT15 cell lines were purchased from the Cell Bank of Chinese Academy of Sciences (Shanghai, China). The three cell lines were respectively grown in McCoy’s 5 A medium, minimun essential medium or RPMI 1640 medium containing 10% fetal bovine serum. Cell passage was carried out in a clean platform. After digestion and centrifugation, the cells were resuspended with complete medium, planted on cell culture dishes or plates and cultured in a humidified atmosphere containing 5% CO_2_ at 37 °C.

### Cell viability

Cells were plated into 6-well plates overnight. After replacement with fresh complete medium, cells were treated with appropriate concentrations of HSP90 inhibitors or in combination with PL for 24 h. Cell viability was measured by trypan blue dye exclusion assay. CI values were determined using CompuSyn software [57]. The CI value <1 indicates a synergistic effect.

### Western blot analysis

Cells and tumor tissues were lysed by using appropriate lysis buffer (AR0101/AR0103, Boster, China), and total proteins were extracted. After determination of protein concentration by the Coomassie Brilliant Blue method, proteins were mixed with protein sample loading buffer (denaturing, reducing, 5×) and then boiled in a boiling bath for 10 min. Samples were separated by SDS-PAGE and then transferred onto PVDF membrane. Next, the membrane was probed with the specific primary antibody overnight. Before probed with the secondary antibody for 1 h, the membrane should be washed with TBST for three times, 5 min each time. High sensitivity ECL substrate kit and ChemiDoc XRS+ imaging system (Bio-rad, USA) were used to detect the signal intensity of the membrane. ImageJ software was used to quantify the signal intensity.

### Measurement of ROS

The cellular ROS levels was measured using the ROS assay kit (Beyotime, Shanghai, China) as previously reported [58]. Cells were plated overnight on coverslips in a 6-well plate, the cells were washed with PBS after treatment with the specified drugs, and then incubated with serum-free medium containing DCFH-DA probe for 30 min. DCFH-DA was hydrolyzed to DCFH and then oxidized to DCF by ROS. Next, the cells were washed three times with serum-free medium, then the coverslips with the cells attached were transferred onto the microscope slides. Fluorescence was visualized using a fluorescence microscope after the cells were washed three times with serum-free medium. DCF in cells can be excited to fluoresce, and the fluorescence intensity of DCF could reflect the level of intracellular ROS. ImageJ software was used to quantify the fluorescence intensity.

### Generation of CHOP knockdown cell line

Recombinant lentivirus targeting CHOP was purchased from GeneChem (Shanghai, China). HCT116 cells were seeded into 12-well plates. Under the guidance of the manufacturer’s protocol, the optimal transfection conditions were obtained by a preliminary experiment. Cells were infected with lentivirus for 12 h, and then replaced with fresh complete medium. The infected cells in the 12-well plate were gradually expanded to 100 mm cell culture dishes. Puromycin was added to the complete medium to select a stable CHOP knockdown cell line for subsequent experiments.

### Immunofluorescence staining

The expression and distribution of γ-H2AX, 53BP1, and CHOP were determined by immunofluorescence staining. Cells were plated on coverslips in 6-well plates and allowed to attach overnight. After treatment the cells in the indicated manner, the drug-treated cells were fixed with 4% paraformaldehyde solution for 15 min, and then rinsed by PBS and permeabilized with immunostaining permeabilization solution (with Triton X-100) for 30 min. This solution includes 0.5% Triton X-100, 20 mM HEPES, 50 mM NaCl, 3 mM MgCl_2_-6H_2_O and 300 mM sucrose. Next, the cells were washed twice with PBS on a shaker for 10 min each time and blocked with 3% bovine serum albumin. Then, the primary antibody was used to probed with the cells on the coverslips overnight at 4 °C overnight. After that, the coverslips with attached cells were washed five times with PBST (phosphate buffered saline with tween 20) for 10 min each time, and then probed with a secondary antibody for 1.5 h at room temperature. Washed the coverslips five times with PBST for 10 min each time again, and the nuclei were stained with antifade mounting medium with DAPI. Immunofluorescence staining was visualized using a fluorescence microscope.

### Comet assay

Prepare a rectangular slide carrier with 0.75% normal melting point agarose gel. Mix the treated cells with 0.5% low melting point agarose, and place them on the previously prepared 0.75% normal melting point agarose gel. Once it coagulates, incubate it overnight in alkaline lysis buffer at 4 °C. Then, incubate it in alkaline electrophoresis buffer for 30 min prior to electrophoresis. Perform electrophoresis for 30 min using 40 V/300 mM parameters on the horizontal electrophoresis instrument. Clean the sample once with ultrapure water and let it air dry before PI staining. Next, observe the sample under a fluorescence microscope.

### Xenograft experiments

HCT116 cells were injected subcutaneously into the flank of athymic BALB/c nude mice (female, five weeks old). The total number of nude mice is set to 24, based on our previous experience. When the tumor volume reached ~100 mm^3^, the mice were randomly divided into four groups with six in each group. Blinding was not performed during the drug treatment process, and the mice were treated with 17-AAG (10 mg/kg), PL (5 mg/kg), or 17-AAG plus PL by intraperitoneal injection once every 2 days. Tumor volumes were calculated according to the formula: *V* = 1/2 × length × width^2^. At the end of the experiment, all mice were euthanized and tumors were harvested, weighed, and frozen for further analysis. All procedures involving animals were performed in accordance with the guidelines of the Institutional Animal Care and Use Committee of Wenzhou Medical University.

### Measurement of MDA

Tumor tissues were lysed by total protein extraction reagent for animal tissues (AR0103, Boster, China), the total protein was extracted and its concentration was determined. According to the kit instructions, TAB storage solution and MDA detection working solution were configured, and standard curves were measured. 200 μL tissue lysate (200 μL PBS for the control group) and 100 μL MDA detection working solution were mixed and heated in a boiling water bath for 15 min. The samples were centrifuged at 1000 rpm for 10 min after cooling to room temperature. Then the absorbance of the supernatant was measured and the content of MDA was calculated.

### Statistical analysis

Data were analyzed using GraphPad Prism 6.0 software. The data were presented as mean ± SEM. *T* test was used to determine the significance of the differences. *P* < 0.05 was considered statistically significant.

## Data Availability

The data presented in this study are included in the article material.
